# Flexible head-casts for high spatial precision MEG

**DOI:** 10.1016/j.jneumeth.2016.11.009

**Published:** 2017-01-30

**Authors:** Sofie S. Meyer, James Bonaiuto, Mark Lim, Holly Rossiter, Sheena Waters, David Bradbury, Sven Bestmann, Matthew Brookes, Martina F. Callaghan, Nikolaus Weiskopf, Gareth R. Barnes

**Affiliations:** a Wellcome Trust Centre for Neuroimaging, Institute of Neurology, University College London, London, UK; b Sobell Department for Motor Neuroscience and Movement Disorders, University College London, London, UK; c Chalk Studios Ltd, 14 Windsor St., N1 8QG, London, UK; d Sir Peter Mansfield Magnetic Resonance Centre, School of Physics and Astronomy, University of Nottingham, Nottingham, UK; e Department of Neurophysics, Max Planck Institute for Human Cognitive and Brain Sciences, Stephanstrasse 1a, 04103 Leipzig, Germany

**Keywords:** Magnetoencephalography, Head localization, Spatial resolution, MRI-MEG Co-registration, Head movement minimization, Head-cast, 3D printing

## Abstract

**Background:**

In combination with magnetoencephalographic (MEG) data, accurate knowledge of the brain’s structure and location provide a principled way of reconstructing neural activity with high temporal resolution. However, measuring the brain’s location is compromised by head movement during scanning, and by fiducial-based co-registration with magnetic resonance imaging (MRI) data. The uncertainty from these two factors introduces errors into the forward model and limit the spatial resolution of the data.

**New method:**

We present a method for stabilizing and reliably repositioning the head during scanning, and for co-registering MRI and MEG data with low error.

**Results:**

Using this new flexible and comfortable subject-specific head-cast prototype, we find within-session movements of <0.25 mm and between-session repositioning errors around 1 mm.

**Comparison with existing method(s):**

This method is an improvement over existing methods for stabilizing the head or correcting for location shifts on- or off-line, which still introduce approximately 5 mm of uncertainty at best (Adjamian et al., 2004; Stolk et al., 2013; Whalen et al., 2008). Further, the head-cast design presented here is more comfortable, safer, and easier to use than the earlier 3D printed prototype, and give slightly lower co-registration errors (Troebinger et al., 2014b).

**Conclusions:**

We provide an empirical example of how these head-casts impact on source level reproducibility. Employment of the individual flexible head-casts for MEG recordings provide a reliable method of safely stabilizing the head during MEG recordings, and for co-registering MRI anatomical images to MEG functional data.

## Highlights

• We propose a method for constructing flexible head-casts to stabilize the head during MEG scanning.
• Co-registration error is minimized by using MRI images to pre-define fiducial coil locations.
• Within- and between-session movement is <0.25 and <1 mm respectively.
• This enables high reproducibility of source level results.

## 1 Introduction

In theory, the spatial precision attainable with magnetoencephalography (MEG) increases monotonically with increasing signal strength (Gross et al., 2003, Hillebrand and Barnes, 2005, Hillebrand and Barnes, 2003). In practice however, this increase is difficult to achieve. Two of the main limitations are errors in co-registration between functional MEG data and anatomical magnetic resonance imaging (MRI) data, and head movement during scanning. Both introduce, at best, ∼5 mm of uncertainty about the location of the head relative to the sensors (Adjamian et al., 2004, Gross et al., 2013, Ross et al., 2011, Singh et al., 1997, Stolk et al., 2013, Whalen et al., 2008). Critically, both sources of error non-linearly compromise the forward modelling accuracy (Hillebrand and Barnes, 2011, Hillebrand and Barnes, 2003), and reduce the signal-to-noise ratio (SNR) through topographical blurring (Medvedovsky et al., 2007, Uutela et al., 2001).

Although some progress has been made in minimizing co-registration error (Hironaga et al., 2014, Koessler et al., 2011, Nunez and Silberstein, 2000, Whalen et al., 2008), for example by stabilizing the head during recording (Adjamian et al., 2004, Singh et al., 1997), or compensating for movements both during and after recording (Medvedovsky et al., 2015, Medvedovsky et al., 2007, Nenonen et al., 2012, Stolk et al., 2013, Uutela et al., 2001), implementation problems have remained. The sources of residual error include misalignment of surfaces, amplification of small placement errors at the front of the head to large errors at the back of the head, and/or reliance on invariance in fiducial placement within and across experimenters and subjects (Adjamian et al., 2004).

Using 3D printing to create solid head-casts which are moulded to the surface of the head internally and to the inside of the MEG scanner externally, we recently showed reduction of co-registration errors to <2 mm (Troebinger et al., 2014a, Troebinger et al., 2014b). Although these first solid head-casts gave access to much higher quality data by minimizing both co-registration error and head movement, they covered the eyes and their rigidity reduced participant comfort, particularly for long recording sessions. Here, we present a new head-cast prototype made of flexible polyurethane foam which leaves the eyes uncovered, and is easier, safer, and more comfortable to use. The improved user comfort is primarily because of the flexibility which makes it easier and faster to get into and out of the MEG scanner helmet (dewar). Furthermore, the 3D printing is now based on an MRI image (as opposed to an optical scan used in Troebinger et al., 2014a, Troebinger et al., 2014b) which both maximises the accuracy with which the cast fits the head, and minimizes co-registration error by predefining the MEG fiducial coil locations in MRI space. We describe the construction pipeline, the within- and between-session head movement for subjects wearing these head casts, and assess the estimated co-registration error. We then show how these improvements give rise to high between-session reproducibility at source level.

## 2 Materials and methods

This section is divided into two parts. First, we describe the methods used for building head-casts. Next, we describe the scanning procedures for evaluating the head-casts with respect to head stabilization, co-registration, and spatial precision.

### 2.1 Participants

Data were collected from four healthy adult subjects (4 men, mean age 32.3 years old). All subjects were right-handed and had no history of neurological or psychiatric disease. One (fifth) participant was excluded from the analysis because of recording errors. Informed written consent was given by all subjects prior to scanning and the experiments were carried out after obtaining ethical approval from the University College London ethics committee (ref. number 5833/001).

### 2.2 MRI data acquisition

**Fig. 1:**
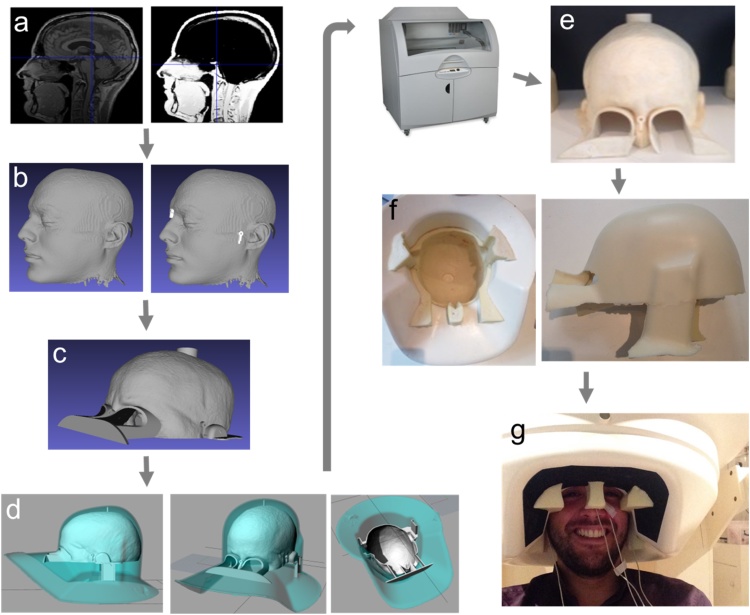
Overview of head-cast construction steps. **a**) Head surface is extracted from an anatomical MRI image using the standard SPM12 segmentation procedure. **b**) Head surface extraction is converted to a surface file and fiducial coils are added. The coil locations are defined in MRI coordinates. **c**) A positioning cylinder at the top of the head is added to the virtual model to define the position of the head inside the head-cast. Eye extensions are added to enable vision during use. **d**) Using and adjusting the positioning cylinder, eye extensions and ear extensions, the virtual head model is positioned appropriately inside a virtual copy of the MEG dewar. **e**) The positive head model is 3D printed. **f**) The 3D print is placed inside the manufacturer-provided dewar copy (as in d) and foam resin is poured in to fill the gap between the printed positive head model and the dewar. The fiducial coil protrusions on the 3D printed head result in coil-shaped and coil-sized indentations in the head-cast (the nasion coil protrusion is visible between the eyes in **e**). **g**) The subject can now wear the flexible foam head-cast and enter into the (real) MEG dewar for scanning.

In order to construct the head-cast, an accurate image of the scalp surface is required. To get this, we first scanned participants in a magnetic resonance imaging (MRI) system (Fig. 1**a**). Images were acquired using a Siemens Tim Trio 3T system (Erlangen, Germany). During the scan, the participant lay in the supine position with their head inside a 12-channel coil. Acquisition time was 3 min 42 s, plus a 45 s localizer sequence. We were very cautious of skin distortions as any such errors could potentially make the head-cast ill-fitting and therefore uncomfortable. For this reason, participants were not given padding or ear phones, as these could displace the skin on the face, head or neck. To minimize audible noise they were instead given ear plugs. The short acquisition time minimizes motion and potential consequential distortions. We used an radiofrequency (RF) and gradient spoiled T_1_ weighted 3D fast low angle shot (FLASH) sequence with the following acquisition parameters: image resolution 1 mm^3^ (1 mm slice thickness), field-of view set to 256, 256, and 192 mm along the phase (A–P), read (H–F), and partition (R–L; second 3D phase encoding direction) directions respectively. Susceptibility differences existing at air-tissue interfaces can lead to magnetic field inhomogeneity and subsequent distortions or signal loss in the acquired image. Therefore, to preserve brain morphology we used a single shot approach with high readout bandwidth (425 Hz/pixel) and minimum echo time (2.25 ms). Consequently no significant geometric distortions were expected or observed in the images. A short repetition time (7.96 ms) was used to minimise acquisition time while the excitation flip angle was set to 12° to ensure sufficient signal-to-noise ratio for the resulting anatomical image. To accelerate the acquisition, a partial Fourier (factor 6/8) acquisition was used in each phase-encoded direction.

### 2.3 Head-cast construction

First, we extracted the scalp surfaces from the MRI data using SPM12 (http://www.fil.ion.ucl.ac.uk/spm/) (Fig. 1**a**). This consists of registering the MRI image with a tissue probability map (Ashburner and Friston, 1997) and classifying tissues into different classes (such as grey matter, skull, skin, etc) on a voxel-by-voxel basis. This is done by constructing a generative model which takes into account both the voxel-specific prior probability of belonging to a given tissue class, and its intensity in the MRI image. This model also estimates and corrects for the bias field (Ashburner and Friston, 2005). We used the skin tissue probability map and converted this into a surface using the MATLAB function ‘isosurface’. We then converted this tessellated surface into standard template library (STL) format (Fig. 1**b**) commonly used for 3D printing. To specify the shape of the fiducial coils, we used optical white light scanning to obtain a 3D representation of a single coil. This was digitally drawn in 3D and then checked for its accuracy both against the digital white light scan as well as the physical coil, using digital measuring callipers. Next three copies of this virtual coil were placed, as per convention, at the nasion, left peri-auricular (LPA), and right peri-auricular (RPA) sites. Note that this was not strictly necessary as any set of distant scalp locations would have enabled the co-registration procedure. This approach therefore does not suffer from inaccuracies in determining anatomical landmarks, as is commonly the case when placing fiducial coils on the head during MEG data acquisition. One constraint on the placement of the coils was ensuring that the coil-body and extruding wire were flat against the scalp, in order to remove unnecessary stress or movement of the coil when the head-cast was put on or taken off.

The original design (Troebinger et al., 2014b) was altered so as to now include eye-hole extensions, ear flaps which extend down below the ears, and a top spacing-cylinder to accurately position the positive head model in the dewar-helmet (Fig. 1**c**–**f**). The ear flaps facilitate getting into and out of the scanner more easily and safely (see *Safety Procedures* for more details) and also provide an external reference of when the head-cast is touching the top of the dewar. The virtual 3D model was thus placed inside a virtual version of the scanner dewar-helmet (Fig. 1**d**) such that the distance to the sensors was minimized (by placing the head as far up inside the dewar as possible) while ensuring that vision was not obstructed. Next, the positive head-model (plus spacing elements and coil protrusions) was printed using a Zcorp 3D printer with 600 × 540 dots per inch resolution (Fig. 1**e**). The 3D printed head model was then placed inside the manufacturer-provided replica of the dewar-helmet and liquid resin was poured in between the surfaces to fill the negative space. The resin expands and sets within ∼30 s, and the resulting flexible foam constitutes the subject-specific head-cast (Fig. 1**f**). Note that the coil protrusions on the 3D print now become indentations in the foam head-cast. The fiducial coils can thus be placed inside the resulting indentations and the head-cast can be worn for scanning (Fig. 1**g**). This removes inaccuracies in determining anatomical landmarks for fiducial placement, and also ensures that the same location is used for repeated scans.

### 2.4 MEG scanning

MEG recordings were made using a 275-channel Canadian Thin Films (CTF) MEG system with superconducting quantum interference device (SQUID)-based axial gradiometers (VSM MedTech, Vancouver, Canada) in a magnetically shielded room. The data collected were digitized continuously at a sampling rate of 600 Hz. We refer to *Safety Procedures* for a description of the general operating and safety procedures.

### 2.5 Experiment 1: between-session variability

We first tested how consistently subjects could be repositioned within the MEG scanner by asking them to reposition themselves in the scanner 10 times. In addition to measuring absolute location of the head-cast using the fiducial coils, we also placed a reference coil on one side of the nose to measure relative displacements between the head-cast and head. Each subject performed 10 separate 10 s trials. For each run, the subject first positioned themselves inside the scanner with the head-cast on, sat still for 10 s, before and after which the fiducial coils were localized, and the subject then exited the scanner and removed the head-cast. This removal and replacement was repeated 10 times.

In addition to the healthy subjects, we also performed a similar experiment using the manufacturer provided spherical current dipole phantom. This experiment was done in order to get an approximation to the system-based noise inherent in localization of the fiducial coils and for comparison with the head-cast results. We did not have a head-cast for the phantom but kept the four fiducials fixed on the surface of it using tape. To mimic the re-positioning, we physically shifted its location between the 10 s trials.

### 2.6 Experiment 2: within-session variability and button presses

To assess the effect of reduced head motion (increasing SNR) and improved head repositioning (improving repeatability), we analysed data from a single subject (subject 3 from Experiment 1) performing button presses across twelve 15-min sessions with 180 trials each. These sessions were spread over four days (which were separated by several weeks) with three runs per day. On each trial, we presented a set of dots moving either left or right, serving as an instruction cue for the subject to indicate the movement direction with a button press upon a subsequent Go signal. All responses were obtained from the right hand. MEG data were acquired at a sampling rate of 1200 Hz.

Data were epoched around the button press onset (time 0), and a beamformer covariance matrix constructed based on the data from the beta band (15–30 Hz) from −2000 to 2000 ms. To extract the source locations, beamformer-based volume-of-interest (VOI) analysis was then carried out, comparing two time windows ([−1500–−1000] versus [500–1000] ms) to generate a statistical chi square volume centred on the average left primary motor cortex peak (−34, −30, 52 mm in MNI space) with a 20 mm radius and 1 × 1 × 1 mm^3^ grid resolution. The data were subsequently smoothed with a full-width half-maximum kernel of 8 mm.

## 3 Results

### 3.1 Between-session movement

**Fig. 2:**
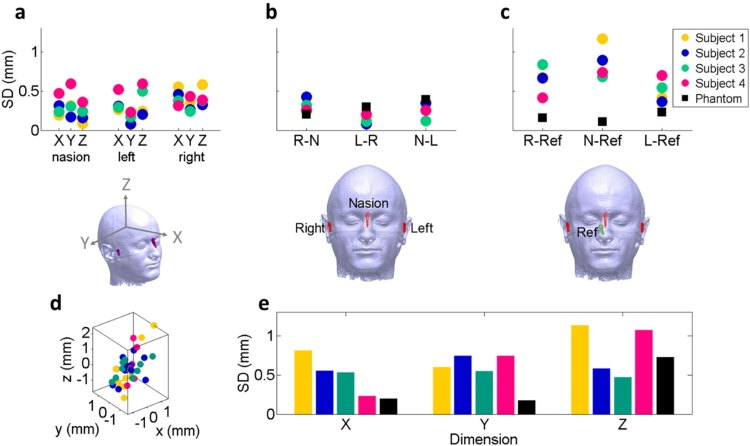
Between-session head movement results from Experiment 1 (re-positioning trials where each of the four subjects came out of the scanner, removed the head-cast, put it back on and re-entered 10 times). **a**) Variability of absolute coil locations. Dots show the standard deviation of the absolute coil location over the course of the experiment. Repositioning is precise to within <0.6 mm standard deviation for any coil in any dimension. **b**) Coil-coil distance variability. The standard deviations are calculated from the distances between the fiducial coils measured in Experiment 1. The distances vary <0.5 mm which is within the range of measurement error, as illustrated by the phantom measurements (black squares). **c**) Reference coil-standard coil distance variability. Same format as b, but based on the distances between each of the three standard fiducial coils and a reference coil placed on the nose. There is more variability with normal subjects than the phantom. **d**) Scatter plot showing absolute locations of reference coil in head-centred (standard coil-defined) space. This plot illustrates dimensions along which the reference coil location varies relative to the standard coils: mostly in the Z dimension (up-down). **e**) Location of the reference coil in head-centred space. Bars encode standard deviation of absolute position of the reference coil in head-centred space measured across 10 repositioning trials. The location of the reference coil deviates <1.2 mm from the fiducial coils in the worst case. Note that variability along the Z dimension is also relatively high with the phantom. The standard deviation over all subjects was 0.50, 0.57, and 0.80 mm for the X, Y and Z dimensions respectively.

To first establish how reproducible the absolute head position was when using head-casts, we measured the fiducial coil locations across 10 repositioning trials (Experiment 1). We found that it was possible to reposition the fiducial coils relative to the MEG system within 0.6 mm standard deviation in any one dimension (Fig. 2**a**).

Next we were interested in whether there is a risk of the coils moving with respect to each other when the head-cast is taken on and off. We examined this by calculating the standard deviation of the distances between fiducial coils across repositioning trials. We found no such measureable effect as the standard deviations of the distances were similar to the standard deviation of the absolute locations (Fig. 2**b**). We found that when we repeated the experiment using a phantom (with the coils fixed on the surface hereof), we observed a similar level of variability, suggesting that this error is due to uncertainty in the (MEG system’s) localization of the coils themselves and not to coil movement.

Since the fiducial coil locations are recorded by the MEG system, changes in head-position relative to the dewar during recording, although undesirable, can be accounted for. A more pernicious source of error is relative movement of the head with respect to the head-cast. To address this directly, we placed a reference coil on the nose of the subject in order to measure the distances between this reference and the standard fiducial coils (Fig. 2**c**). Unlike with the previous analysis where there was no difference between measurements made with the phantom and normal subjects, we now observed an effect beyond measurement error. We found that the variability in the location of the head-cast relative to the head was predominantly due to uncertainty in the Z dimension of 1.2 mm standard deviation.

Next we were interested in whether these differences in distances to the reference coil could be attributed to differences in location along some spatial dimensions more than others. Fig. 2**d** shows that the most variable dimension is the Z (up-down) dimension. Fig. 2**e** shows the standard deviation of the reference coil with respect to ‘head-centred’ space, meaning that the coordinate frame is defined by the three standard fiducial coils. These values reflect how much the reference coil moved around relative to the standard fiducial coils inside the head-cast in X (front-back), Y (left-right), and Z (up-down) dimensions. We thus found that the main axis along which additional variance occurs is the Z (up-down) axis (Fig. 2**d**,**e**). Surprisingly, we found this highest variation in the Z dimension to be true for both phantom and human measurements. This suggests increased measurement uncertainty in this plane, which may be unrelated to the head-cast but perhaps due to the MEG sensors and algorithms used to localise the coils, or simply the vertical movement of the scanning chair (on which the phantom rested) over time.

### 3.2 Within-session movement

**Fig. 3:**
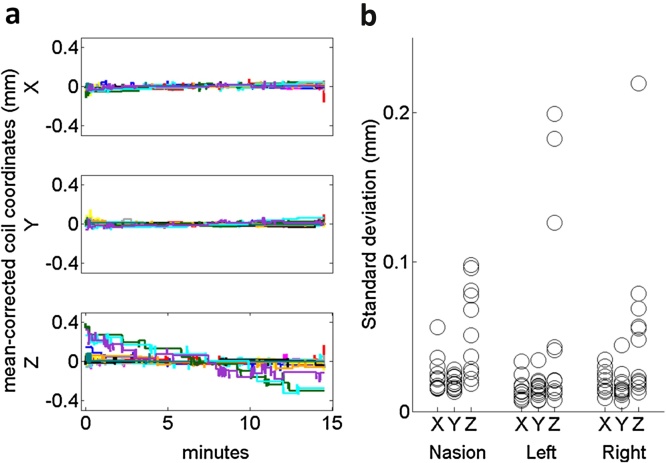
Within-session head movement. Data from Experiment 2. **a**) Absolute location of the left coil in the X, Y and Z dimensions over the course of 12 (colour coded) 15-min trials. The location is mean-corrected individually for each trial. We find that the variability across time is negligible. The largest movements are downwards (from positive to negative) in line with the subject sliding down in the chair. **b**) Circles show the standard deviations of the absolute coil locations for all 12 trials in all dimensions and for all coils. The standard deviation of the locations recorded was 0.22 mm at maximum. Z (vertical) is consistently the most variable dimension.

To evaluate the head location stability over time, a single subject was scanned on 12 separate trials lasting 15 min each (Experiment 2). We found that results were almost identical across fiducial coils. For any coil, relative movements over twelve 15-min runs were sub-millimetre (<0.75 mm) and the movement predominantly occurred as drift in the vertical direction (left coil shown as an example, Fig. 3**a**). Note that these traces were mean-corrected (such that the average head position over each 15 min period was set to zero) but that the standard deviations of these means were 0.25, 0.25 and 0.26 mm for the X, Y and Z dimensions respectively. Across all coils, we found the standard deviations of locations over time to be below 0.22 mm for any coils in any dimensions (Fig. 3**b**). The maximal absolute changes in the coil locations were 0.69, 0.5 and 0.75 mm for the left, nasion, and right fiducial respectively (the corresponding minimal changes were 0.06, 0.11, and 0.06 mm). All of the maxima were in the Z (up-down) dimension. We reason that the explanation for the slightly larger absolute changes and standard deviations in this dimension is that the height of the head-cast inside the dewar may change slightly over the course of a trial, e.g. because the subject relaxes and therefore slouches and loses posture more. We also suspect that there is slightly lower sensitivity in the Z axis (see phantom data in Fig. 2**e**) which could be due to the sensor configuration (see Discussion).

### 3.3 Data reproducibility

**Fig. 4:**
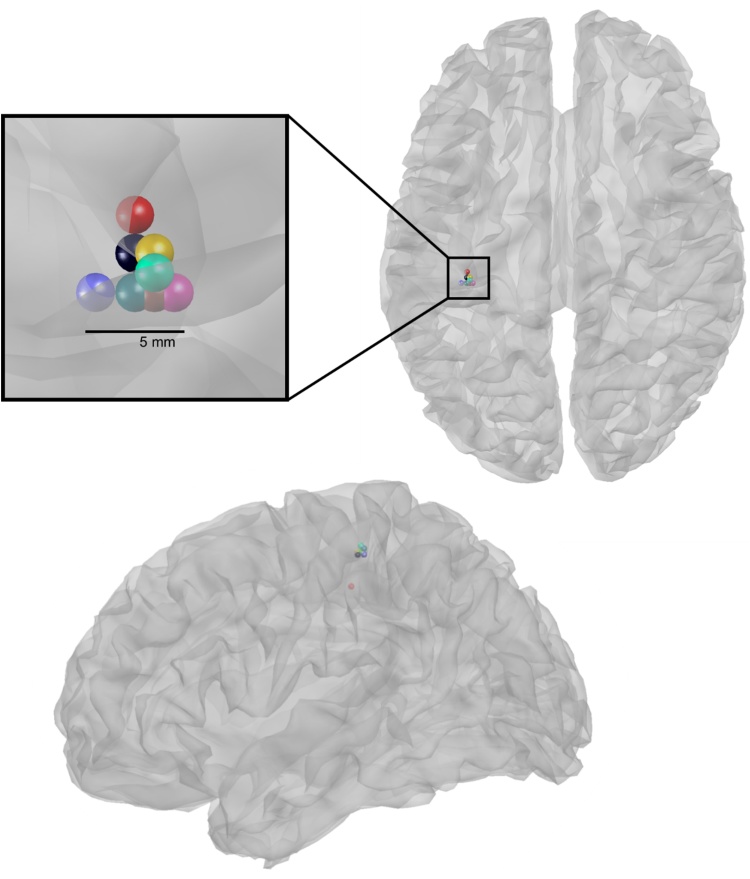
Consistency of data features across four separate scanning days. Coloured spheres represent beta (15–30 Hz) rebound peaks from Experiment 2. The peak locations reflect the maximum chi square statistic when comparing pre-button press data (−1500–−1000 ms) to post-button press data (500–1000 ms) across a 20 mm radius, 1 × 1 × 1 mm^3^ resolution sphere centred around the average left primary motor cortex peak (−34, −30, 52). Note that the solutions were not constrained by the mesh as reconstruction was volumetric. Data shown are smoothed using an 8 mm kernel.

In Fig. 4 we show recordings from a single subject performing repeated right hand button presses over multiple sessions conducted over several days (Experiment 2). The beamformer peak from 11/12 sessions (consisting of 180 trials each) fell on the same three 1 mm^3^ grid locations while one fell more dorso-laterally when constrained to the same contralateral hemisphere as the others.

## 4 Discussion

We have developed a novel method for building flexible and subject-specific MEG head-casts to stabilise the head during recording. This method makes use of the subject’s MRI image both to build the head-cast by 3D printing an image of the head shape, and to co-register the MEG and MRI data. We find that using this technique for head-cast design, the within-session head movement measured with a single subject recorded over several sessions ranges from 0.06 to 0.75 mm when measured over an approximately 15 min period. We estimate the maximal co-registration error during these measurements to be around 1.2 mm.

The head-casts were designed to improve both subject comfort and safety. By making the casts flexible and adding ear flaps, we made it easier to enter and exit the dewar, minimizing the risk of getting stuck or requiring assistance. Additionally, we added eye holes which enable subjects to see and therefore participate in experiments using visual stimuli and/or eye tracking. Together, these features make the head-casts less intimidating to wear and open up the possibility of a wider range of experiments. Importantly the head-casts do not obstruct breathing, vision, or talking although hearing may be mildly compromised.​ We have not found these head-casts to induce anxiety or claustrophobia. However, we screened for these by asking subjects about claustrophobic vulnerability prior to any testing.

The other major difference between this generation of head-casts and the previous, is that the 3D print is now based directly on the MRI image eliminating the need for optical scanning. We optimised an MRI acquisition sequence to eliminate distortions on the surface of the head. The manufacturing process is nonetheless not completely straightforward. Whilst some head-casts fit very well, others require removal of sections that constitute pressure points on the head, typically near the eye holes and temples. This seems to be more pronounced in subjects with longer hair.

With respect to the MRI imaging sequence used, our requirements in selecting the optimal sequence were as follows: 1) to have moderately high resolution (1 mm isotropic), with a large field of view in order to be able to distinguish the scalp with sufficient spatial specificity; 2) to maximise SNR efficiency, and 3) to minimise any artefact that might appear outside of the brain. The most common source of uncontrollable artefact in MRI is participant motion. Focal motion during data acquisition leads to distributed artefact outside of the brain, which it was essential that we avoided by keeping the sequence as short as possible. However, acceleration options were limited since we did not want to use parallel imaging to accelerate the acquisition, as this approach inherently leads to aliasing that needs to be unfolded. Therefore, we chose to focus solely on FLASH imaging which could achieve these requirements. Since it has no preparation pulses (unlike MPRAGE-like acquisitions), it can continuously acquire data with very short TR (7.96 ms here) giving it high SNR efficiency with a minimal scan time of just 3 min 42 s. Piloting showed that this approach was more than adequate for MEG head-cast design.

A key point is that the head-casts can be used to maintain the head in any position – based on the positioning of the digital scalp outline within the dewar - even when that means that over small regions there is no foam between the head and the dewar (and the distance to the sensors is minimized). But this of course also means that this particular positioning of the head will not be optimal for all experiments. It is worth pointing out that the distance cylinder length varies as a function of head size; the smaller the head, the more offset from the top is needed to ensure vision.

With respect to the subjective experience of wearing the head-cast, we find that subjects experience them as constraining and unusual at first, but that they quickly get accustomed to the experience (usually after a few recordings), and they improve at entering and exiting the dewar efficiently. Multiple subjects have remarked that it is obvious to them when the head-cast is fit incorrectly when entering the dewar, but not necessarily before. We have also observed that some experienced subjects find it easier to relax while being scanned when wearing a head-cast as they do not have to minimize or inhibit movement. This is an important improvement, as previous methods have relied on self-stabilization (e.g. with bite bars to hold the head in position (Adjamian et al., 2004, Muthukumaraswamy, 2013, Singh et al., 1997)) which induces a risk of increased muscle activity and concomitant artefacts (Kumar et al., 2003, Muthukumaraswamy, 2013, O’Donnell et al., 1974, Whitham et al., 2007).

The main advance of this head-cast approach is that unlike other co-registration minimization approaches, the specification of fiducial points, and extraction of scalp surface based on the same original MRI scan simultaneously minimizes co-registration error and head movement. In turn, this improves the reproducibility of data (Fig. 4). In previous work (Troebinger et al., 2014b) we have shown that the reduction of within-session movement from 5 to 1 mm gives rise to an effective 5 fold increase in SNR. Notably, high reproducibility implies high precision but not necessarily accuracy. However, the high SNR recordings mean that this framework can be used to directly test between different forward models (e.g. the head in different positions, see (López et al., 2012)) delivering an accuracy measure that encompasses the complete source reconstruction pathway.

A number of caveats remain. First, we address the increased uncertainty of coil localisation in the Z dimension as observed with increased error in phantom measurements (Fig. 2**e**). This could either be due to the internal algorithm used to locate the fiducial coils based on their magnetic signature or simply the movement of the scanner-chair. Second, the co-registration estimate based on the reference coil (Fig. 2**c**) may have been pessimistic as the tape holding the reference coil in place on the side of the nose extended beyond the coil and was easily tugged on by the head-cast. Additionally, the location of the reference coil was both below and outside of the dewar, meaning that it would provide a further challenge to the internal MEG coil localization procedure. Moreover, prospective motion correction methods where a small optical marker is tracked with sub-micron movement and sub-degree rotation precision has shown that placing the marker on the bridge of the nose is unstable, as uncorrelated movement between the marker and the brain can be observed, likely due to malleability of the skin (Todd et al., 2015).

As mentioned perhaps the most pernicious source of error due to these devices is movement of the subject’s head relative to the head-cast. In this case the fiducial locations would appear stable over time whilst, for example, the subject was slowly slipping out of the cast. Based on our reproducibility measurements in Fig. 2**c** the refitting of the cast over time does not seem to be a problem, but there may be some subjects (due to the shape of their heads) who can slide downwards within the headcast without head-cast movement. In the future we will begin using a 4th coil (attached to the head) for more routine measurements in order to quantify this.

Given that the brain is suspended in corticospinal fluid inside the skull, it must be acknowledged that it remains ambiguous whether the difference between the brain location while supine (during the MRI scan) and sitting (during the MEG scan) could be affecting our estimates. There is a risk that when the head changes orientation with respect to gravity, the brain shifts when the density or thickness of the CSF layer between the brain and the skull changes. It has been approximated that the this change in thickness is ∼30% which equates to approximately 1 mm (Hill et al., 1998, Rice et al., 2013). We emphasize however that using head-casts while subjects are supine removes the ability to use gravity to exit the dewar, causing the safety to be compromised. Although it would be interesting to directly quantify these shifts though such comparisons, we decided not to due to the safety issues outline below.

Other potential data acquisition problems which we posit that the head-casts solve to a degree but which we have not formally tested are to muscle artefacts (Muthukumaraswamy, 2013), particularly when using bite-bars (Adjamian et al., 2004), and slow within-session drifts (Stolk et al., 2013).

Moreover, we have extended the prototype design such that it can accommodate subject with long or thick hair (Supplementary Fig. 1). This extends the usefulness of these devices and means that a larger segment of the population can be scanned. We are working on testing whether this modification affects head stabilization, re-positioning, or in any way introduces unknown errors.

The results of the present study suggest that employment of the individual flexible head-casts for MEG recordings provide an accurate and reliable method of safely stabilizing the head location during MEG recordings, and for co-registering MRI anatomical images to MEG functional data. This design is ideally suited for studies which require sensitive longitudinal MEG measurements.

## 5 Safety procedures

Any head-casts pose a significant source of risk of injury to subjects if used incorrectly. Because the head-casts are designed to fit the subject’s head internally and the MEG dewar externally, the participant’s head is firmly fixed inside the dewar during scanning. This means that any unexpected movement of the chair or MEG system has the potential to cause severe neck injury. Our primary safety measure is therefore to ensure that neither the chair nor the dewar is moved while the subject is wearing a head-cast. This means that the initial positioning of the subject (as well as any subsequent adjustments to the height or angle of the chair) only takes place when the subject is not inside the scanner wearing a head-cast. To enter or exit the dewar, the subject therefore slides in and out of the seat unassisted. In our experience, this takes some practice but is easily and quickly mastered. However, this means that only healthy, agile volunteer subjects are suitable for head-cast scanning. In order to ensure maximal comfort and safety of participants, we have developed a set of safety procedures to be followed by all researchers carrying out MEG scans involving head-casts. We also screen subjects to avoid scanning participants with claustrophobia, and place a panic button inside the magnetically shielded room should the subject wish, at any time, to stop scanning.

We advise that only authorised personnel are allowed to scan volunteers with a head-cast.

For these reasons we have decided never to use the head-cast with a subject in supine position where the consequences of unexpected relative movement between the dewar and the bed could be much more serious.

We refer to our safety guidelines, standard operating procedures, training guide, volunteer guide, and emergency procedures available on the MEG community website (http://megcommunity.org/ under instrumentation > peripherals > subject stabilization) which also contains a link to an instruction video for experimenters.

## Conflict of interest

We have read the Society for Neuroscience’s policy on conflict of interest and declare the following interests: ML holds a position in a company (Chalk Studios Ltd) that produces the head-casts reported in this work; he took part in the design and manufacture of the head-casts but played no part in the data analysis. The other authors declare no competing financial interests.
