# Supplementary material for: Flexible head-casts for high spatial precision MEG
Source: J Neurosci Methods. 2017 Jan 30;276:38–45. doi: 10.1016/j.jneumeth.2016.11.009 (PMC5260820; doi:10.1016/j.jneumeth.2016.11.009)
Supplement: Supplementary file 1 [file mmc1.docx]

**Supplement**


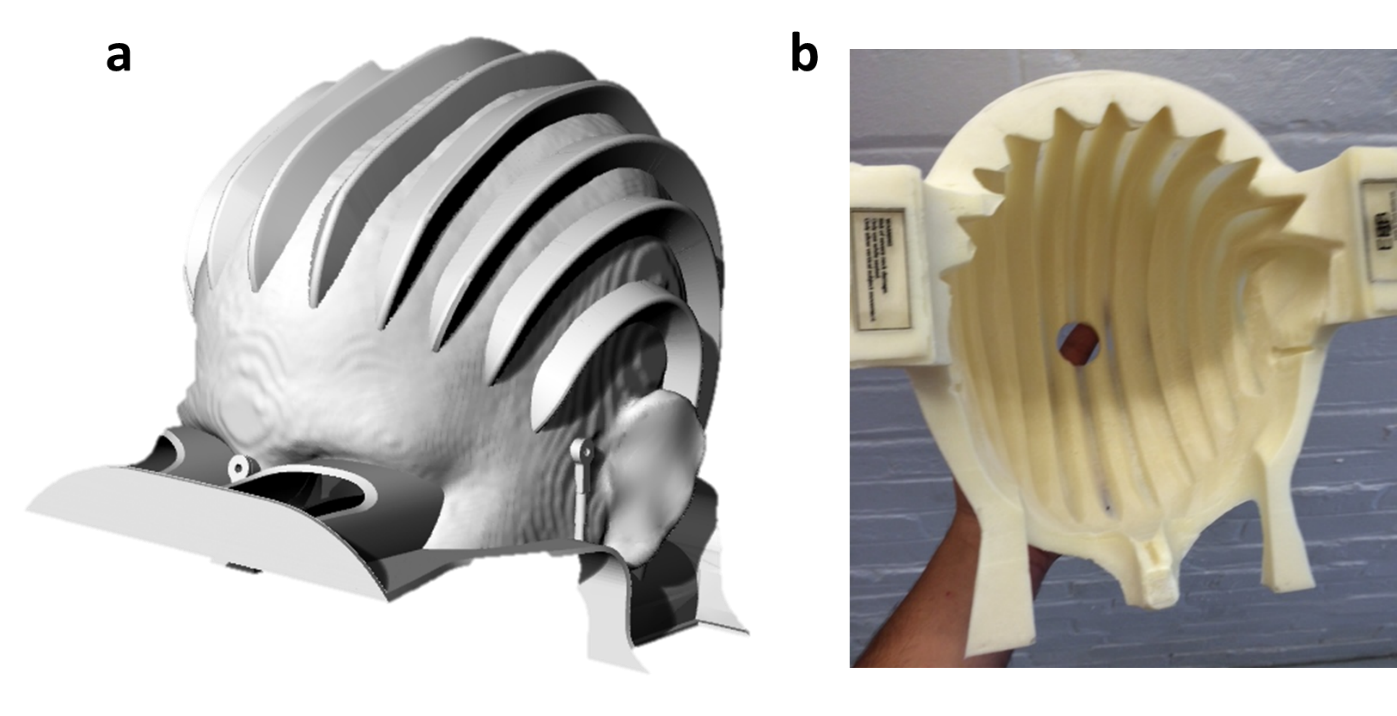


**Supplementary Figure 1**: Head-cast design modified to accommodate hair. **a**) Virtual head model of a subject with grooves to accommodate hair volume. The MRI scan is insensitive to hair so protrusions are added to the scalp extraction to create space. **b**) Photograph of head-cast designed to accommodate hair.
